# Raman Molecular Fingerprints of Rice Nutritional Quality and the Concept of Raman Barcode

**DOI:** 10.3389/fnut.2021.663569

**Published:** 2021-06-23

**Authors:** Giuseppe Pezzotti, Wenliang Zhu, Haruna Chikaguchi, Elia Marin, Francesco Boschetto, Takehiro Masumura, Yo-Ichiro Sato, Tetsuya Nakazaki

**Affiliations:** ^1^Ceramic Physics Laboratory, Kyoto Institute of Technology, Kyoto, Japan; ^2^Department of Orthopedic Surgery, Tokyo Medical University, Tokyo, Japan; ^3^The Center for Advanced Medical Engineering and Informatics, Osaka University, Osaka, Japan; ^4^Department of Immunology, Graduate School of Medical Science, Kyoto Prefectural University of Medicine, Kyoto, Japan; ^5^Department of Dental Medicine, Graduate School of Medical Science, Kyoto Prefectural University of Medicine, Kyoto, Japan; ^6^Laboratory of Genetic Engineering, Kyoto Prefectural University, Kyoto, Japan; ^7^Research Center for Japanese Food Culture, Kyoto Prefectural University, Kyoto, Japan; ^8^Experimental Farm, Graduate School of Agriculture, Kyoto University, Kizugawa, Japan

**Keywords:** Raman, barcode, rice, nutrients, quality, fingerprint, molecular

## Abstract

The nutritional quality of rice is contingent on a wide spectrum of biochemical characteristics, which essentially depend on rice genome, but are also greatly affected by growing/environmental conditions and aging during storage. The genetic basis and related identification of genes have widely been studied and rationally linked to accumulation of micronutrients in grains. However, genetic classifications cannot catch quality fluctuations arising from interannual, environmental, and storage conditions. Here, we propose a quantitative spectroscopic approach to analyze rice nutritional quality based on Raman spectroscopy, and disclose analytical algorithms for the determination of: (i) amylopectin and amylose concentrations, (ii) aromatic amino acids, (iii) protein content and structure, and (iv) chemical residues. The proposed Raman algorithms directly link to the molecular composition of grains and allow fast/non-destructive determination of key nutritional parameters with minimal sample preparation. Building upon spectroscopic information at the molecular level, we newly propose to represent the nutritional quality of labeled rice products with a barcode specially tailored on the Raman spectrum. The Raman barcode, which can be stored in databases promptly consultable with barcode scanners, could be linked to diet applications (apps) to enable a rapid, factual, and unequivocal product identification based on direct molecular screening.

## Introduction

The need for a reliable method capable of screening the nutritional characteristics of food emerges from a growing customers' interest (and concerns) about food composition, processing, and dietetic impact (1). In the case of rice, and of cereals in general, the genetic basis and breeding have a significant impact on grain quality and nutritional value. Although customers might perceive the rice quality as a context-specific and heterogeneous attribute (2), nutritional facts represent objective features. Accordingly, the availability of reliable nutritional information should enable consumers to make cognizant choices, irrespective of their heterogeneous criteria of selection (3). Nowadays, available gene-based markers enable the selection/accumulation of specific gene alleles that impact on preferred nutritional traits and grain quality. This approach includes tailoring specific grain proteins and amino acids (4, 5), enriching with vitamins and specific minerals (6), controlling glycemic impact (7), and boosting up antioxidant characteristics through enhanced fractions of phenolic and flavonoid compounds (8). As modern genomic practices increasingly lead to multiplication of rice varieties and on-demand hybrids, the customers' need for rapidly accessible and reliable information grows and stimulates the development of data-driven scientific databases and related accessibility apps (9).

The presently available analytical methods for evaluating the nutritional characteristics of rice include: gas chromatography for locating differential metabolites (10, 11), polymerase chain reaction techniques for determining primary amino acid sequences (12, 13), proteome analysis based on protein sequencing or mass spectrometry (14), and amylose-iodine colorimetric tests for the characterization of polysaccharides (15). Despite the popularity of those methods, however, none of them meets the demand for *in situ*, rapid, and non-destructive analyses. The Raman spectroscopic method in principle meets all those requirements. In addition, the Raman method enables multiple analyses within a single measurement, thus allowing the concurrent and highly efficient evaluation of a range of key nutritional targets (16–23). However, such a multivalent information is encrypted in complex spectral features, whose deconvolution and quantitative interpretation require specifically constructed algorithms, whose development is yet in its infancy. Provided that such algorithms could become available, Raman spectroscopy could allow for contactless biochemical analyses with no sample pre-treatment or homogenization needed, no extraction, and no labeling agents required.

In this study, we discuss the application of Raman spectroscopic analyses to *in situ* assessment of the nutritional characteristics of rice. We shall specifically discuss analytical algorithms for the determination of amylopectin and amylose concentrations, phenolic compounds and grain protein contents, which directly represent the glycemic, antioxidative, and nutritional characteristics of rice cultivars, respectively. These algorithms are then applied to six different Japanese rice cultivars specifically selected to cover a wide spectrum of structural characteristics. In addition, we propose the storage of the detected Raman characteristics in a database accessible with apps by means of a specially tailored Raman code. This code is a machine-readable representation of the Raman spectrum and thus contains objective molecular-scale information, which specific apps (barcode readers) might translate into users' readable information. The advantage of the Raman barcode with respect to a conventional barcode is that the information contained in the former, once appropriately certified, could serve as factual and data-driven source for customers, while also providing science-consolidated branding strategies to producers.

## Experimental Procedures

### Rice Samples

**Figure 1:**
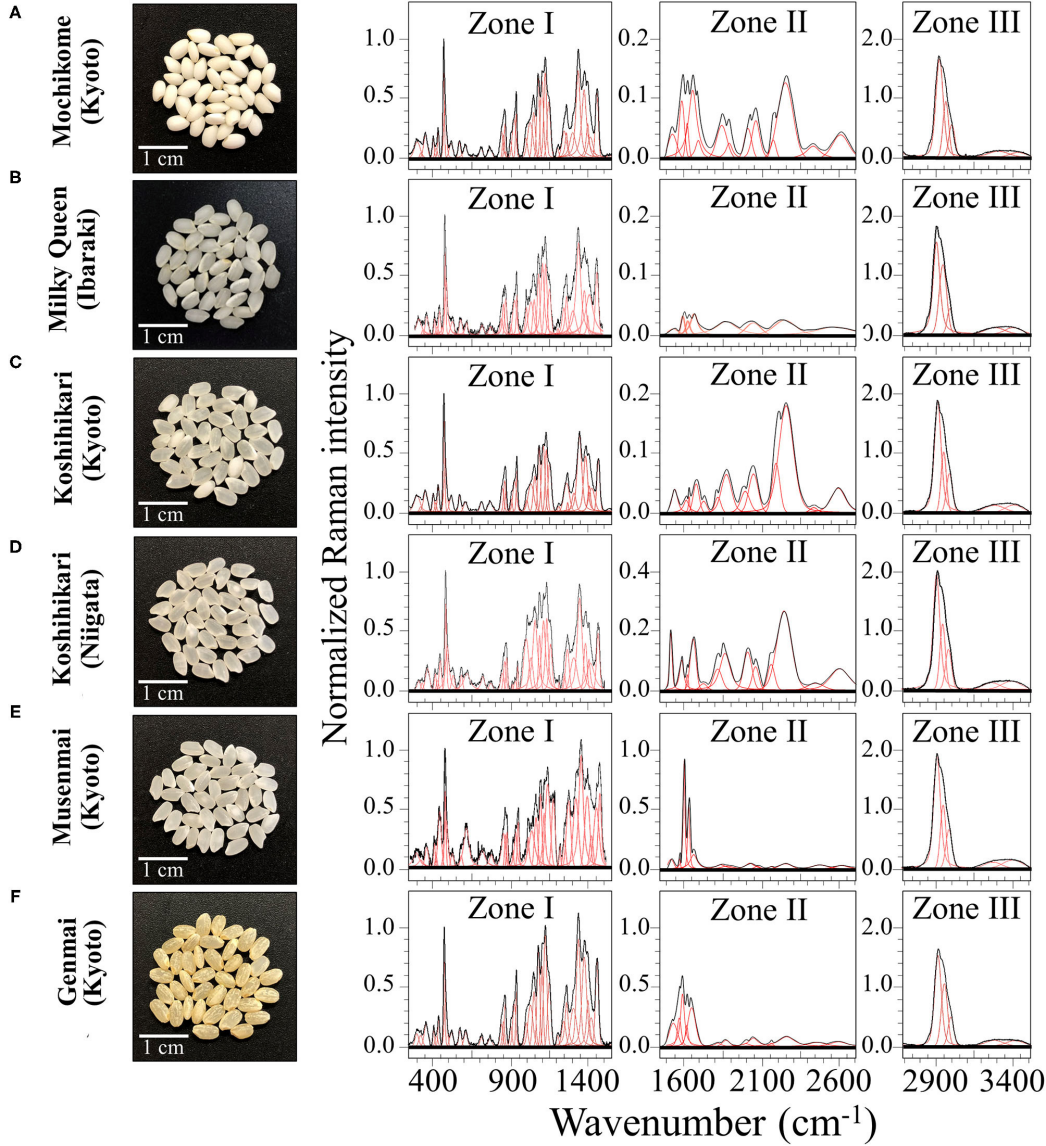
Laser microscopy images of kernel cross sections from six different Japanese rice cultivars **(A)** Mochikome (Kyoto), **(B)** Milky Queen (Ibaraki), **(C)** Koshihikari (Kyoto), **(D)** Koshihikari (Niigata), **(E)** Musenmai (Kyoto), and **(F)** Genmai (Kyoto). Photographs of the kernels of six popular Japanese rice cultivars (cf. insets) and their related Raman spectra (divided into three separate spectral regions referred to as Zone I, II, and III at 250–1,500 cm^−1^, 1,500–2,700 cm^−1^, and 2,700–4,500 cm^−1^, respectively). Spectral deconvolution into Voigtian sub-bands is drawn with red lines.

Six popular rice cultivars were studied; photographs of the rice kernels are shown in Figure 1. One cultivar of the six investigated ones was glutinous rice (mochigome) with high adhesiveness characteristics (Figure 1A). Produced in Kyoto Prefecture, this glutinous rice is “Shinhabutaemochi” harvested in Kyoto. According to a discriminant analysis based on X-ray diffraction data published by Zhang et al. (24), this type of glutinous rice belonged to a specific group of crystallinity (referred to as Group I) with a relatively high content of monoclinic phase in its amylose structure.

One low-amylose cultivar referred to as “Milky Queen” (Figure 1B) was harvested in Ibaraki Prefecture. This is a relatively new cultivar (registered in 1995), which represents a mutant line derived from NMU treatment of Koshihikari egg cell. The Milky Queen cultivar has been reported to have a grain superior to that of Koshihikari in terms of cooking, eating, and processing quality, although its major agronomic traits were nearly equivalent to those of Koshihikari in paddy fields (25). The major agronomic traits of Milky Queen are conspicuously equivalent to those of Koshihikari in paddy fields.

Two “Koshihikari” cultivars from two different Prefectures, namely, Kyoto (Figure 1C) and Niigata (Uonuma type) (Figure 1D), belonged to the most widely grown type of rice in Japan since more than 50 years. As a general characteristic, the superior eating quality of the Koshihikari type with its sticky and chewy texture has made its widespread popularity in Japan. Koshihikari brands cultivated in different places are reported to possess structural characteristics that significantly vary depending on the climate of their regions of provenience (26). The Niigata Uonuma is perhaps the most famous type of Koshihikari rice due to the very favorable climate conditions, but the Koshihikari Kyoto has gained popularity for its peculiar difference in texture and its flavor, which is retained even after long storage times.

The product name referred here as “*Musenmai Kyoto*” (Figure 1E) was also Koshihikari; it was harvested in Kyotango city in the northern part of Kyoto Prefecture. The term “*Musenmai*” simply indicates that it is pre-washed rice.

Finally, the sample in Figure 1F, referred to as “Genmai,” was also Koshihikari cultivated in Kyoto. Unpolished rice has recently gained popularity in Japan as a healthy food since the outer bran retains vitamins and minerals that are usually removed by polishing.

All the investigated samples were harvested in the year 2018 and kept into sealed vacuum bags in a dark storage cabinet at 20% humidity and a constant temperature of 20°C. The Raman investigations, conducted as described in the next section, did not require specific manipulations of the rice samples, except for one cross-section experiment specifically designed to obtain structural differences between the core and the shell of individual kernels. Cutting was performed with a hand-held diamond coated surgical blade to obtain a good surface finishing with a limited plastic deformation.

### Raman Spectroscopic Experiments

Raman spectra were systematically collected on grain samples of six different rice cultivars. No homogenization, addition of labeling agents or other manipulations was made on the samples. Spectra were collected using a dedicated spectroscope (T-64000, Horiba/Jobin-Yvon, Kyoto, Japan) operating in microscopic confocal mode with a 20 × optical lens. A triple-monochromator was used for high-resolution spectral acquisitions (better than 0.1 cm^−1^). The blue line of a 488 nm Ar-ion laser (Stabilite 2017, Spectra Physics, Mountain View, CA, United States) was used as an excitation source and applied with a power of 10 mW. The Raman light was diffracted into a monochromator connected with an air-cooled 1,024 × 256 pixels charge-coupled device (CCD) detector (CCD-3500V, Horiba Ltd., Kyoto, Japan). The acquisition time for one spectrum was 30 s. Average of 30 spectra collected on 30 different grains for each sample were analyzed after being deconvoluted into Voigtian sub-bands using commercially available software (LabSpec 4.02, Horiba/Jobin-Yvon, Kyoto, Japan). All spectra were normalized to the glucose ring signal at 478 cm^−1^. Spectra were first represented in Origin 6.0 (Microcal Software Inc., Northampton, MA, United States) subtracted of their background, and then smoothed with the FFT Filter tool. The first derivative of the noise-free (smoothed) functions was computed and wavenumbers at maxima retrieved upon analytically setting the derivative functions to zero. The penetration depth of the laser probe into rice kernel samples could be assessed by means of the probe-response function procedure using the defocusing method (27). The Raman probe penetration depth did not significantly vary among the tested samples and was typically in the order of 0.2–0.3 mm for glutinous, unpolished, and polished rice kernels. To observe the samples in cross-section, a small laboratory clamp was used to support the grain.

## Results

### Raman Spectra of the Investigated Rice Cultivars

Figures 1A–F show low-resolution Raman spectra collected on as-purchased kernels of six different rice cultivars (cf. photos and labels) in the spectral region 250–3,500 cm^−1^. The spectra presented in Figure 1 represent averages of all 30 spectra acquired per each type of cultivar. For visualization purposes, the collected (average) spectra were then divided into three main frequency intervals, as follows: a low frequency Zone I (between 250 and 1,550 cm^−1^), a middle frequency Zone II (between 1,550 and 2,700 cm^−1^), and a high frequency Zone III (between 2,700 and 3,500 cm^−1^). Raman components from different spectral zones were deconvoluted into Voigtian sub-bands (also shown in Figure 1) to fit the experimental spectra.

Zone I is mainly dominated by signals from polysaccharides with bands below 500 cm^−1^, at 950–1,200 cm^−1^, and at 1,200–1,500 cm^−1^, being specific for skeletal breathing modes, coupling CC and CO symmetric stretching modes, and CH deformation modes, respectively (28). A strong band in this zone was located at ~478 cm^−1^ and related to glucose ring stretching. This glucose ring-stretching signal was selected as a reference to normalize the overall spectrum of all three Zones, because it preserved a constant morphology and showed negligible shifts in frequencies for different rice samples. The relative fraction of different polysaccharides, specifically amylose and amylopectin in rice kernels, covers a fundamental role in the glycemic impact, which greatly varies among different cultivars (29). We thus dedicated specific efforts here (cf. forthcoming section Raman Fingerprints of Polysaccharide Structures) in locating a suitable Raman algorithm capable to quantitatively characterize the fractional amounts of different polysaccharides in rice.

Zone I also contained strong signals from amino acids, including phenylalanine (benzene ring breathing at ~1,004 cm^−1^) and tryptophan (indole ring breathing, H-scissoring, and CH_2_-related vibrations at ~756, 875, and 1,360 cm^−1^, respectively) (30). Given the importance of the above two aromatic amino acids both as precursors of secondary metabolism (5) and as nutrients for human health (31), we conducted specific calibrations in order to construct Raman algorithms capable of quantitatively assess their fractional amounts in rice kernels. These algorithms will be presented in the forthcoming section Raman Assessments of Phenylalanine and Tryptophan Contents and the related calibrations in the Supplementary Material.

An important feature of Zone II is the Amide I signal (cf. Figures 1A–F). This is a composite vibrational signal combining C=O and C-N stretching, and N-H bending modes, all occurring within the so-called amide plane of the protein structure. The Amide I spectral zone, which is comprised between 1,620 and 1,700 cm^−1^, is usually deconvoluted into four sub-bands characteristic of the secondary structure of proteins. Sub-bands representing α-helix, random coil, β-sheet, and β-turn are typically reported at frequencies 1,638, 1,652, 1,668, and 1,701 cm^−1^, respectively (32, 33), with the 1,638 cm^−1^ band possibly overlapping with the C=C-C vibration in lignine (18). According to the relative (areal) intensity of the above sub-bands, the Raman spectral profile of the Amide I signal has been used for quantifying the secondary structure of proteins (34). Detailed analyses of protein structure in rice kernels based on the above notions will be presented in section Raman Analyses of Protein Structures.

In Zone II, a series of additional bands were detected in the region 1,900–2,800 cm^−1^ (cf. Figures 1A–F; Zone II). Such bands cannot easily be explained according to the intrinsic/natural structure of rice kernels. They included signals at 2,150–2,165 cm^−1^, which possibly arise from C=C=O bond stretching in ketene or relate to N=N=N bond stretching in azide. Ketene dithioacetals (III) are organic sulfur compounds used in Japan as an agricultural fungicide and quite effective for rice blast control (35). Conversely, sodium azide has been successfully utilized to generate genetic variability in rice breeding (36). Isocyanate, thiocyanate, and isothiocyanate are also added as insecticides and/or to enhance stress tolerance to abiotic stress, plant growth, and plant yield (37); the above synthetic compounds display relatively strong and broad bands at ~2,260 cm^−1^ (N=C=O bond stretching), 2,150 cm^−1^ (S-C=N bond stretching), and 2,050 cm^−1^ (N=C=S bond stretching), respectively. We indeed observed such bands in the Raman spectrum of various cultivars (cf. Figures 1A–F; Zone II), as discussed in detail in section Cross Section Experiments on Protein-to-Carbohydrate Ratio.

Zone III presented the strongest Raman signals detected in all collected spectra, which appeared in the spectral region between 2,800 and 3,050 cm^−1^. These strong signals likely arose from polysaccharides and partly from lipids, but could also be contributed by synthetic compounds. The sub-region 2,850–2,980 cm^−1^ typically presented two distinct bands characteristic of symmetric and antisymmetric H–C–H stretching vibrations in methyl and methylene groups. In addition, the stretching vibrations of–C–H and =C–H groups appeared with Raman band centered at ~2,900 and >3,000 cm^−1^, respectively (38). Weaker and broad signals were also detected at higher frequencies (3,150–3,500 cm^−1^). These signals are likely related to symmetric and antisymmetric stretching of NH_2_ bonds, which could be assigned to a number of different molecules containing amine functional groups (39). In Zone III, no significant differences in the main peaks could be detected among the different cultivars investigated. However, some subtle differences appeared, which will be discussed in a later section.

### Raman Fingerprints of Polysaccharide Structures

**Figure 2:**
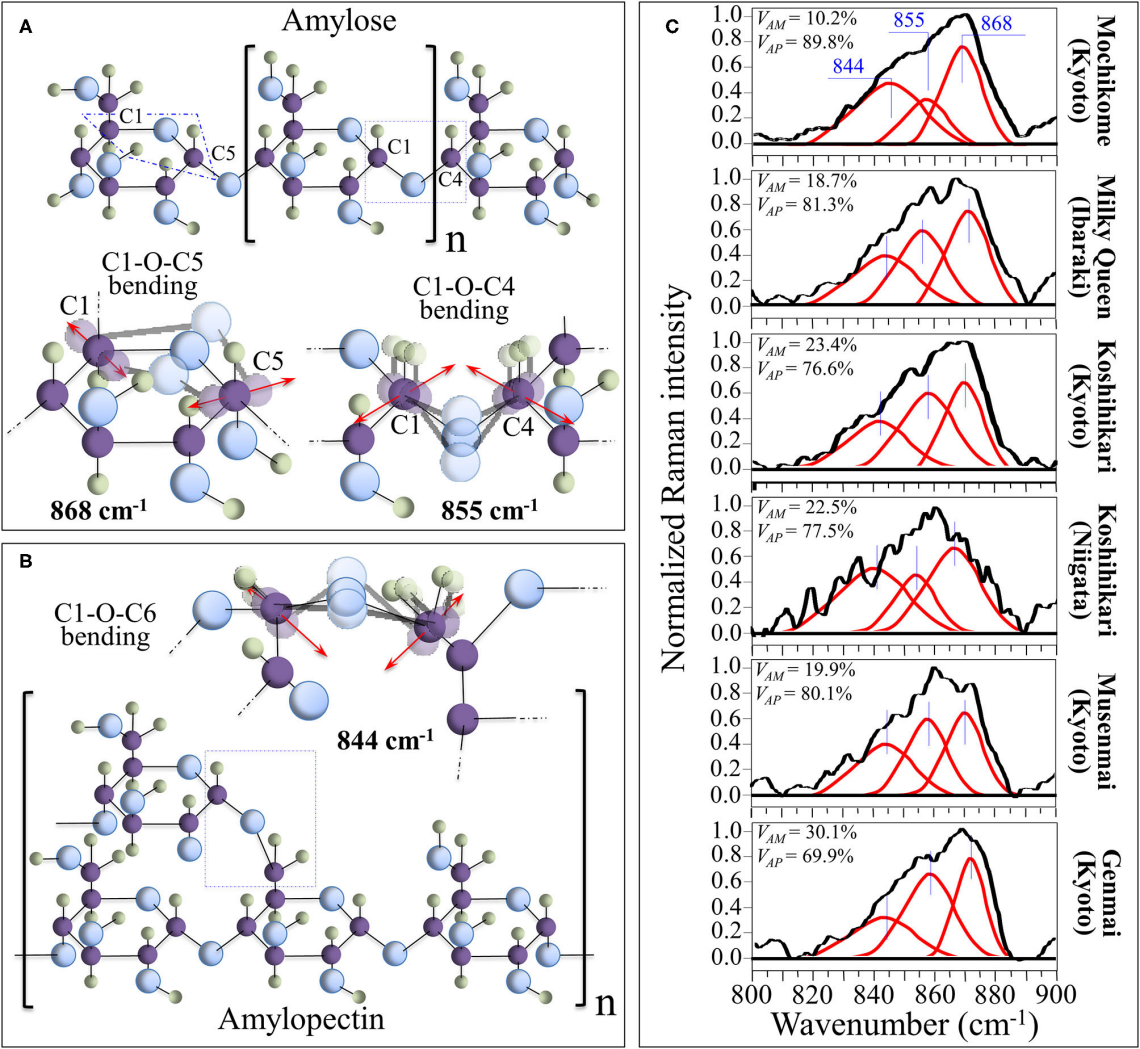
Schematic drafts of amylose **(A)** and amylopectin **(B)** structures, presented with emphasis placed on C-O-C bending modes, whose characteristic Raman frequencies are given in inset. In **(C)**, deconvoluted Raman spectra of different rice cultivars in the spectral region 800–900 cm^−1^ are shown (cf. labels) with the calculated volume fractions of amylose and amylopectin given in inset.

Morphological differences in the spectral region 830–895 cm^−1^, which represents the C-O-C bending mode, can be exploited for quantitatively assessing amylopectin and amylose volume fractions. Figures 2A,B show schematic drafts of amylose and amylopectin structures, respectively, with emphasis placed on C-O-C bending modes. As shown in these figures, the Raman sub-bands centered at 868, 855, and 844 cm^−1^ can be assigned to bending modes of C1-O-C5 (within rings), C1-O-C4 (at chain linkages), and C1-O-C6 (at branching points; in amylopectin only), respectively. Deconvoluted Raman spectra of different rice cultivars in the spectral region 800–900 cm^−1^ are shown in Figure 2C (cf. labels). A comparison among these spectra showed clear differences in the relative intensity of the three deconvoluted sub-bands. More specifically, the relative intensity of the bending vibration at chain linkage (C1-O-C4 bending at 855 cm^−1^) and that at branching points (C1-O-C6 bending at 844 cm^−1^) were the lowest and highest, respectively, in the Mochikome type of rice, while they also substantially varied among different cultivars. Since the Raman signal at 844 cm^−1^ is only peculiar to amylopectin, it is possible to set an algorithm to locate the percent volume fraction of amylose, *V*_AM_ (%), from the relative intensity of Raman sub-bands, as follows: (40).

(1) $$\begin{array}{l} V_{AM}\left(\%\right)=\frac{\alpha -R_{P}}{\alpha +R_{P}\left(\beta -1\right)}\times 100 \end{array}$$

(2) $$\begin{array}{l} R_{P}=\frac{I_{844}}{I_{844}+I_{855}+I_{868}} \end{array}$$

in which *R*_*p*_ is defined as the Raman polysaccharides ratio and *I* represents the integrated areas of the sub-bands with maxima at the frequencies given in subscript. The calibration plot and the best fitting curve are shown in Supplementary Figure 1. The Raman calibration procedure was validated by iodine colorimetry method (according to ISO standard) (41) in Ref. 40. The linear correlation between amylose contents measured by the present Raman method and by the iodine colorimetry method on the same cultivars obeyed a high coefficient of determination (*r*^2^ = 0.997).

The numerical calibration constants α and β, which were determined from the best fitting curve as 32.6 and 1.1, respectively, take into account the different cross-sections of the Raman signals (cf. also the description of polysaccharide calibration in the Supplementary Material) (40). Percent volume fractions of amylopectin, *V*_*AP*_(%), were then computed according to the following equation:

(3) $$\begin{array}{l} V_{AP}\left(\%\right)=100-V_{AM}\left(\%\right) \end{array}$$

This method was validated by independent amylose-iodine colorimetry measurements performed on the same samples (40). Quantitative Raman assessments of amylose/amylopectin fractions according to the above Equations (1–3) are given in inset to Figure 2C.

### Raman Assessments of Phenylalanine and Tryptophan Contents

**Figure 3:**
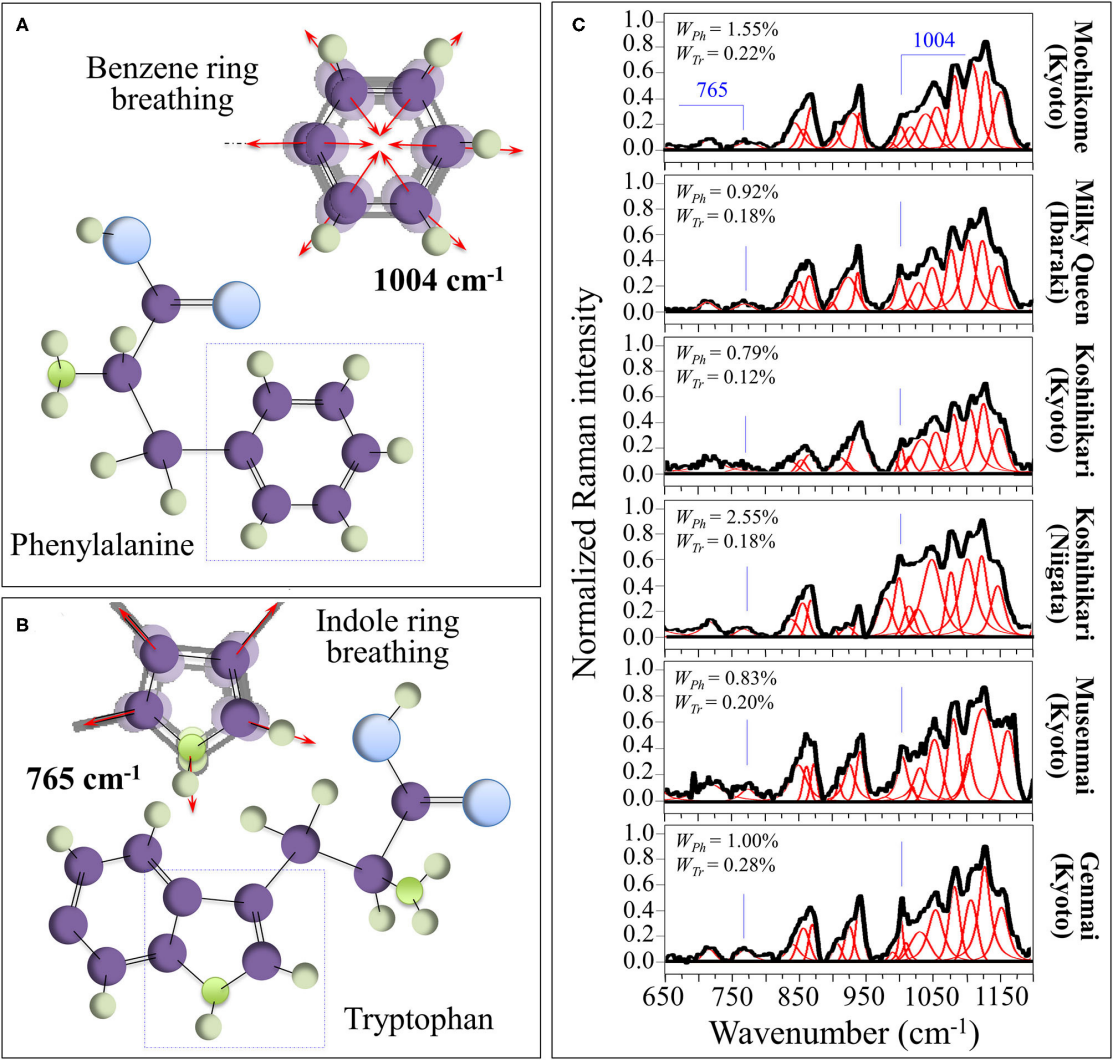
**(A)** Ring breathing vibration in the molecular structure of phenylalanine, which displays at 1,004 cm^−1^ in the Raman spectrum of rice; and, **(B)** the molecular structure of tryptophan and its peculiar indole ring breathing vibration at ~765 cm^−1^. In **(C)**, deconvoluted Raman spectra of different rice cultivars in the spectral region 650–1,200 cm^−1^ are shown (cf. labels) with the calculated weight fractions of amylose and amylopectin given in inset.

The sharp and relatively strong Raman line characteristic of benzene ring breathing in phenylalanine (at ~1,004 cm^−1^) can be used to quantitatively characterize the presence of this aromatic amino acid in rice kernels. A fractional quantification becomes possible provided that accurate calibrations are preliminarily conducted with known fractions in order to produce a viable Raman algorithm. For this purpose, we preliminary performed calibrations in order to develop such algorithm (cf. Supplementary Figure 2A). Figure 3A shows the ring breathing vibration in the molecular structure of phenylalanine, which displays at 1,004 cm^−1^ in the Raman spectrum of rice. In Supplementary Figure 2A, a calibration plot is shown, which relates the weight fraction of phenylalanine, *W*_*Ph*_, to the intensity ratio, *R*_*Ph*_ = *I*_478_/(*I*_478_ + *I*_1004_), between its ring-breathing band and the glucose ring-stretching band at 478 cm^−1^ contributed by all polysaccharides. Accordingly, in the cases of current rice samples, the weight fraction of phenylalanine, *W*_*Ph*_, in the samples obeyed the following equation:

(4) $$\begin{array}{l} W_{Ph}\left(\%\right)=\frac{R_{Ph}^{{\;}^{\prime }}}{\gamma +R_{Ph}^{{\;}^{\prime }}}\times 100 \end{array}$$

(5) $$\begin{array}{l} R_{Ph}^{{\;}^{\prime }}=\frac{I_{1004}}{I_{478}} \end{array}$$

in which *I* represents the integrated areas of the sub-bands with maxima at frequencies given in subscript, while γ ( = 5.47 and 4.40 for mochikome and uruchimai, respectively, as obtained from the calibration curves shown in Supplementary Figure 2A) is a numerical calibration factor that takes into account the different cross-sections of the Raman signals.

Figure 3B shows a similar analysis for the aromatic amino acid tryptophan (cf. calibration plot in Supplementary Figure 2B). The molecular structure of tryptophan and its peculiar indole ring breathing vibration at ~765 cm^−1^ are schematically depicted in Figure 3B, while section Supplementary Figure 2B shows a calibration plot, which relates the weight fraction of tryptophan, *W*_*Tr*_, to the relative intensity, *R*_*Tr*_ = *I*_478_/(*I*_478_ + *I*_765_), of its indole ring-breathing band to the glucose ring-stretching band at 478 cm^−1^ contributed by all polysaccharides. The weight fraction of tryptophan, *W*_*Tr*_, obeyed the following equation:

(6) $$\begin{array}{l} W_{Tr}\left(\%\right)=\frac{R_{Tr}^{{\;}^{\prime }}}{\delta +R_{Tr}^{{\;}^{\prime }}}\times 100 \end{array}$$

(7) $$\begin{array}{l} R_{Tr}^{{\;}^{\prime }}=\frac{I_{765}}{I_{478}} \end{array}$$

in which *I* represents the integrated areas of the sub-bands with maxima at frequencies given in subscript, while δ ( = 7.15 and 17.16 for mochikome and uruchimai, respectively, as obtained from the calibration curves shown in Supplementary Figure 2B) is a numerical calibration factor that takes into account the different cross-sections of the Raman signals.

In Figure 3C, deconvoluted Raman spectra are shown for different rice cultivars (cf. labels) in the spectral region 650–1,200 cm^−1^. This zone is rich in partly overlapping band components whose complete assignment is difficult. However, differences in relative intensities of selected bands from these spectra were rationalized according to Equations (4)/(5) and (6)/(7) and based on the calibration parameters; the respective phenylalanine and tryptophan weight fractions for different cultivars were obtained as shown in insets.

### Raman Analyses of Protein Structures

**Figure 4:**
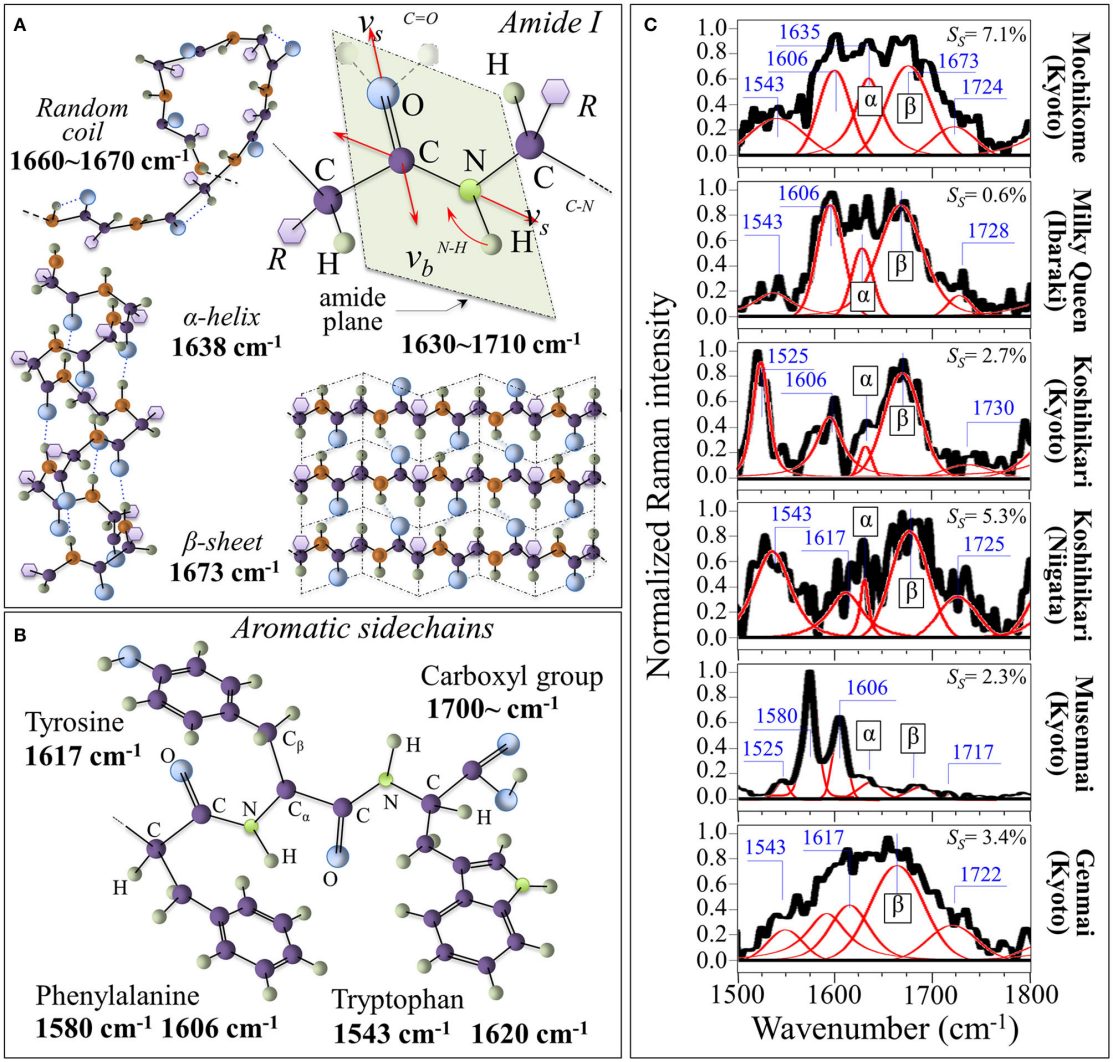
**(A)** Amide I vibrational modes and schematic drafts of the secondary structures of proteins in which it takes place (cf. related Raman frequencies in inset); **(B)** aromatic sidechains of tyrosine, phenylalanine, tryptophan, and carboxyl group (with related Raman frequencies in inset). In **(C)**, deconvoluted Raman spectra in the Amide I region are shown for the six investigated rice cultivars (the α and β labels represent α-helix and β-sheet, respectively). In inset, the fraction of succinylated lysine is shown as computed according to the relative intensity of the band at 1,720–1,730 cm^−1^ (cf. labels).

Figure 4A shows the Amide I vibrational modes, schematic drafts of the secondary structures of proteins in which it takes place, and the related Raman frequencies; Figure 4B depicts aromatic sidechains of tyrosine, phenylalanine, tryptophan, and the carboxyl group (with related Raman frequencies in inset). In Figure 4C, Raman spectra in the Amide I region are shown for the six investigated rice cultivars. The integrated intensity of the sub-bands can be used to give a reasonably accurate estimate of the fractions of protein secondary structures (42, 43). Such fractions present well-known links to specific rice proteins. According to Mawal et al. (44), rice albumin possesses the unique structural characteristic of being predominantly comprehensive of β-sheet and β-bend (reverse turns) structures, with no other rice protein having so far been reported to possess a similar structure. On the other hand, rice prolamin predominantly contain α-helical, while glutelin mainly exhibits a random coil structure with a minor fraction of α-helix secondary structure. According to these notions, one could interpret the morphological differences in the Amide I signals of different cultivars by associating them to different types of proteins. Detailed spectral analyses were focused on the specific spectral interval between 1,500 and 1,800 cm^−1^. This spectral zone was deconvoluted into five sub-bands, which were located with respect to their frequencies (cf. values in inset) and assigned with respect to their vibrational origins. As far as the Amide I vibration is concerned, the Bands in the ranges 1,640–1,658, 1,665–1680, and 1,660–1,670 cm^−1^ are generally assigned to α-helix, β-sheet, and random coil configurations, respectively. These bands have the same physical origin, but they represent different protein secondary structures, as depicted in Figure 4A. We found the strongest signal among the above secondary configurations at ~1,673 cm^−1^ (**β**-sheet) for all types of studied cultivar. In the Musenmai type, a doublet signal at 1,580 and 1,606 cm^−1^ was preponderant over that of the Amide I, which was very weak due to the polishing procedure. A preponderance of the β-sheet signal in the Amide I signal marks the predominance of albumin among other rice proteins. However, as a general feature, the β-sheet band was a quite broad signal. This feature can be explained with assuming the overlap of different β-sheet sub-structures due to the presence of hydrogen bonds at the C=O carboxyl bonding location (cf. Figure 4A). As a matter of fact, it has been reported (45) that decreases in the Amide I frequency to ~1,677 and ~1,665 cm^−1^ occur when for peptide compounds containing one and two hydrogen bonds, respectively.

The explanation of the physical origin of the additional bands observed in the 1,500–1,800 cm^−1^ region is challenging. However, the bands appearing between 1,710 and 1,730 cm^−1^, which are seen in all types of investigated cultivars except for glutinous rice (cf. Figure 4C), have been the main object of a previous study (46). These bands arise from C=O stretching of the succinyl functional groups in lysine, which has been reported for structurally modified starch samples. The areal ratio, *R*_*A*_, of the 1730 cm^−1^ C=O stretching band to the Raman activity from C-C stretching in the interval 890~970 cm^−1^ (namely, the sum of the areas of three band components located at 905, 915, and 943 cm^−1^), *A*_1730_/(*A*_905_ + *A*_915_ + *A*_943_), was related to the degree of succinate substitution, *S*_*s*_, as determined from the titrimetric method in order to obtain a quantitative calibration curve (46). The calibration curves for mochikome and uruchimai rice obeyed the following linear equations [cf. also plots in Supplementary Figure 3 obtained from (46)]:

(8) $$\begin{array}{l} R_{A}=0.55293S_{s}-0.00192(\text{mochikome}) \end{array}$$

(9) $$\begin{array}{l} R_{A}=0.55254S_{s}-0.00184(\text{uruchimai}) \end{array}$$

with a Pearson's *r* factor >0.99 in both cases. Areal ratios were then computed from the experimental spectra and rationalized according to Equations (8) and (9); the respective degrees of succinate substitution are shown in insets to Figure 4C. Living aside the highest *S*_*S*_ value found in glutinous rice, the highest and the lowest degrees of succinylation were found in Koshihikari (Niigata) and Milky Queen (Ibaraki) brands. Lysine succinylation is a protein post-translational modification defined as the transfer of a succinyl group to a lysine residue. According to a recent paper by Zhang et al. (47), the charge alteration of the lysine residue induced by succinylation in turn causes substantial alterations to the chemical properties of proteins, which are important for cellular processes. This point will be discussed in more details in a later section.

The additional Raman bands that could be observed in the frequency interval 1,500–1,800 cm^−1^ (Figure 4C) can be explained by considering specific vibrations of aromatic side chains in proteins. According to Takeuhi (48), the bands seen at 1,580/1,606 cm^−1^, 1,543/1,620 cm^−1^, and 1,617 cm^−1^ can be assigned to phenylalanine, tryptophan and tyrosine side chains, respectively (cf. draft in Figure 4B). The Koshihikari (Kyoto) brand, however, presented a relatively sharp feature a ~1,525 cm^−1^ (cf. Figure 4C), which was clearly separated from the 1,543 cm^−1^ band (common to all other brands), which were assigned to tryptophan. We assigned this band to C=C stretching in carotenoids (49). The assignment to carotenoids will be confirmed in the cross-section experiments described in the next section.

### Cross Section Experiments on Protein-To-Carbohydrate Ratio

**Figure 5:**
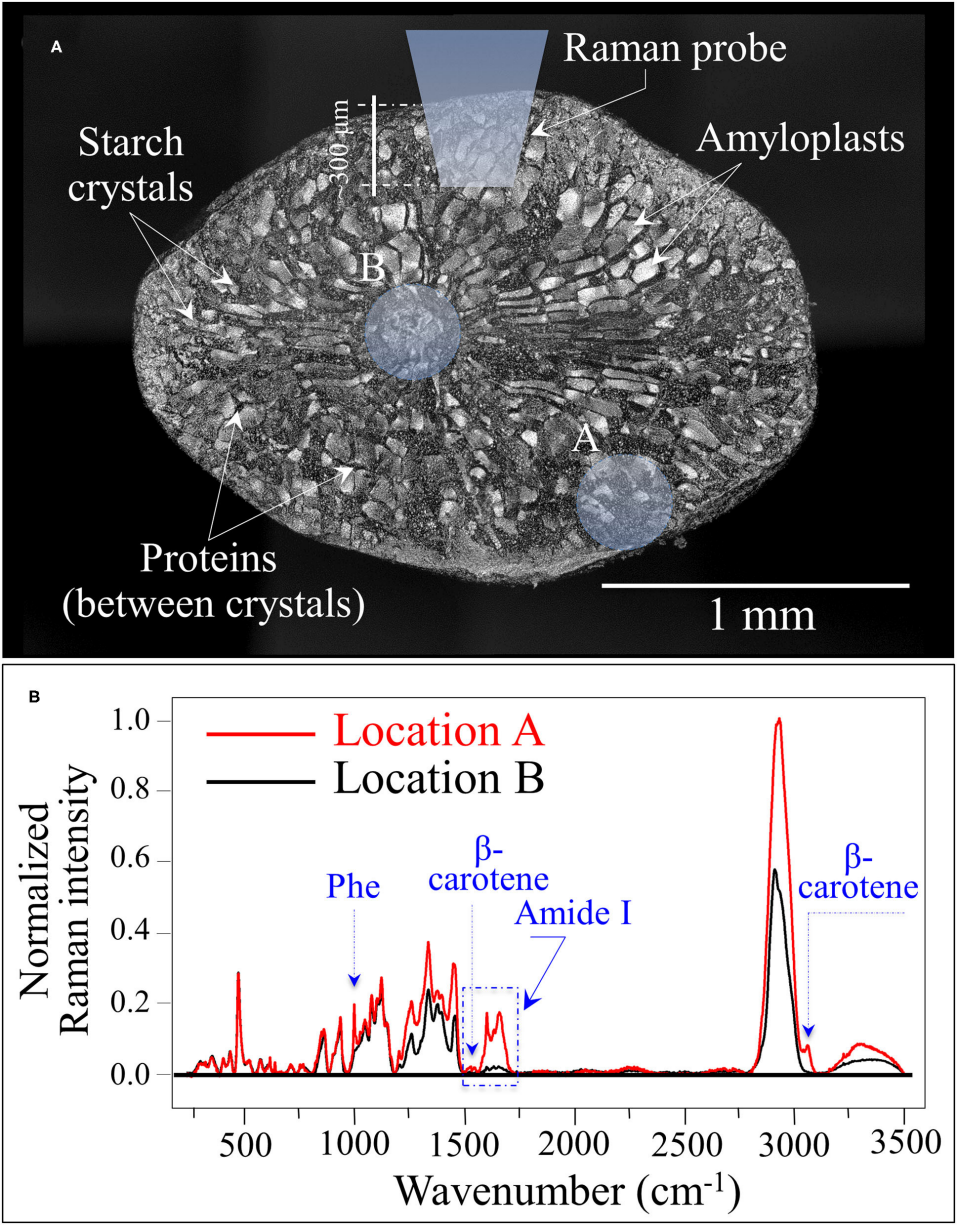
**(A)** Laser micrograph (and structural features given in inset) of the cross section of a Koshihikari (Kyoto) rice kernel; the draft in inset schematically shows the laser penetration depth when the Raman measurement is non-destructively made. The two locations labeled as A and B produced the Raman spectra shown in **(B)**. The three main differences between spectra collected in A and B relate to phenylalanine and Amide I signal intensities, and to an additional band located at ~3,070 cm^−1^ [cf. labels in **(B)**].

Figure 5A shows a laser micrograph (with structural features given in explanatory insets) taken on the cross section of a rice kernel from the Koshihikari (Kyoto) cultivar. An additional draft in inset schematically shows the laser penetration depth when the Raman measurement is made on a non-sectioned rice kernel. Two selected locations for the laser spot are marked on the cross section, peripheral and central to it (labeled as A and B, respectively). The Raman spectra collected at the locations A and B are shown in Figure 5B. Upon normalizing to the glucose ring vibration band at 478 cm^−1^, three main features could be observed from comparing spectra from different locations: (i) the phenylalanine band was significantly stronger at the peripheral in A than in B; (ii) similar to (i), the Amide I signal was ~7 times stronger in A than in B; and, (iii) bands in the high frequency region were generally stronger at Location A but also a new band appeared at ~3,070 cm^−1^. The above three items are assessed hereafter.

**Figure 6:**
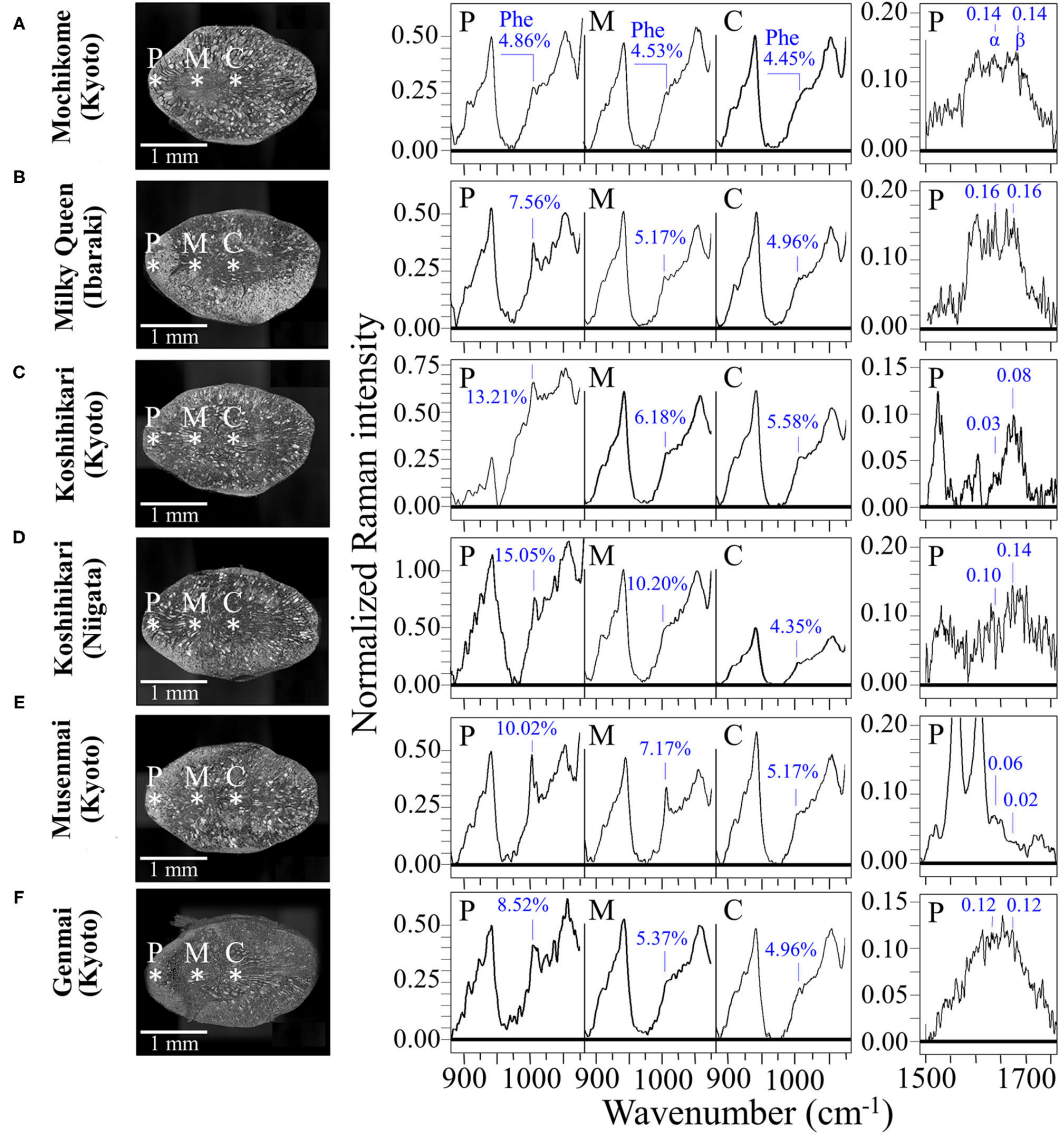
Laser microscopy images of kernel cross sections from six different Japanese rice cultivars **(A)** Mochikome (Kyoto), **(B)** Milky Queen (Ibaraki), **(C)** Koshihikari (Kyoto), **(D)** Koshihikari (Niigata), **(E)** Musenmai (Kyoto), and **(F)** Genmai (Kyoto). Laser microscopy images of kernel cross sections from six different Japanese rice cultivar; in the middle part of the figure, the related high-resolution Raman spectra collected in the spectral region 875–1,075 cm^−1^) at peripheral, middle, and central locations of the sectioned kernels (labeled on the cross-section images with asterisks and P, M, and C, respectively) with the respective weight fractions of phenylalanine shown in inset; on the right side of the figure, high-resolution Raman spectra in the Amide I region as collected in the frequency interval 1,500–1,700 cm^−1^ at the peripheral location P of different types of rice kernels. The respective sums of the relative intensities of signals from α-helix and β-sheet (labeled as α and β, respectively), are given in inset and were used to calculate the protein-to-carbohydrate ratio, *R*_*P*/*C*_.

Figures 6A–F compare the kernel cross sections and the Raman spectra of different rice cultivars as collected at peripheral, middle, and central locations (P, M, and C, respectively; cf. insets to Figure 6). A clearly decreasing trend in phenylalanine content from peripheral toward central locations of the rice kernel was a characteristic common to all the investigated samples, except for the mochikome type (cf. spectra in the region 900–1,100 cm^−1^ in the middle of the figure and the related weight fractions, *W*_*Ph*_, given in inset). However, despite a lack of concentration gradient, the total amount of phenylalanine in glutinous rice was the lowest among the measured types of rice. Conversely, the highest fraction of phenylalanine (*W*_*Ph*_ = 15.05%) was recorded in the Koshihikari (Niigata) cultivar, but it was also accompanied by the highest decreasing gradient toward the center of the kernel. Both types of Koshihikari cultivars (i.e., Kyoto and Niigata) were definitely superior in phenylalanine content to the Milky Queen type, while the lower fractions found for the Musenmai type are likely related to the polishing procedure. Given the importance of phenylalanine as an aromatic amino acid essential as nutrient for human health, recent studies have focused on the modification of biosynthetic pathways for increasing the amount of this amino acid in rice (5, 50), which should thus be considered as an important trait in enhancing the nutritional value of rice (31).

A comparative estimate of the protein content can be obtained by monitoring the protein-to-carbohydrate ratio in rice cultivars through the Raman relative intensity of the sum of Amide I bands related to β-sheet (1,673 cm^−1^) and α-helix (1,635 cm^−1^) (representative of proteins) to the glucose ring stretching band at 478 cm^−1^ (representing the cumulative amount of carbohydrates). This ratio, henceforth referred to as *R*_*P*/*C*_, varied among different cultivars as shown by its values given in inset to Figure 6 (cf. spectral region 1,550–1,800 cm^−1^ on the right side of the figure). This approach is similar to that Ren et al. (51) validated by means of chemical analyses. Those researchers used the ratio between the Amide III band at 1,278 cm^−1^ and the carbohydrate band at 362 cm^−1^ (C-C stretching). We instead selected the stronger signal at 478 cm^−1^ as a reference signal for polysaccharides and the Amide I band instead of the Amide III signal because the latter signal in rice is likely contributed also by lipids. A comparison among the investigated cultivars revealed that the type of rice richest in proteins was the Milky Queen (*R*_*P*/*C*_ = 0.32) followed by the Koshihikari (Niigata) (*R*_*P*/*C*_ = 0.24). Notably, the above former value was higher than that recorded in the Mochikome (*R*_*P*/*C*_ = 0.28). Also the Genmai type contained a relatively high amount of proteins (*R*_*P*/*C*_ = 0.24). On the other hand, the Musenmai type contained one order of magnitude lower amount of proteins (*R*_*P*/*C*_ = 0.08) as compared to the above-mentioned two richest types. The *R*_*P*/*C*_ ratios in the central area of the kernels (not shown) were always weaker by more than one order of magnitude as compared to the respective peripheral values. Since such values were comparable with the signal noise, we did not attempt their measurement.

The high frequency signals in the interval 2,800–3,500 cm^−1^ were significantly stronger when spectra were recorded in the peripheral area of the kernel (cf. Figure 5B). This circumstance can be explained by considering that signals in that zone might reflect the systematically higher amount of proteins recorded in the peripheral zone. However, the strongest band in the overall spectrum, which is centered at ~2,900 cm^−1^, mainly arises from CH in the glucose ring, in addition to CH_2_ and CH_3_ stretching in both proteins and carbohydrates. Conversely, the stronger N-H stretching signal observed between 3,200 and 3,500 cm^−1^ is a clear indication of higher protein content in the peripheral zone of the kernel, because N is not present in carbohydrate structures (52). In the specific case of the Koshihikari (Kyoto) cultivar, an interesting feature systematically appeared at ~3,070 cm^−1^ in spectra from the peripheral zone of the kernel (cf. Figure 5B). This band can be assigned to C-H stretching in the aromatic rings of the β-carotene structure (49, 53). The presence of this relatively weak but well isolated band can be considered as a fingerprint of carotenoids. This observation fits well with the other unique feature of the Koshihikari (Kyoto) cultivar, namely, the relatively sharp feature at ~1,525 cm^−1^ related to β-carotene C=C stretching (as mentioned in the context of Figure 4C). Rice endosperm generally does not contain β-carotene, which represents an important nutritional characteristic because it converts into vitamin A in the human body. As an essential micronutrient in daily diet, β-carotene is seen as a nutritional requirement to counteract vitamin A deficiency in countries where rice is the staple food. Burkhardt et al. (54) was the first who designed an experiment for making the rice endosperm capable of synthesizing β-carotene using the geranyl diphosphate isoprenoid as a precursor in phytoene synthase. Since that successful experiment, the production of the so-called “Golden rice” has become very popular and the procedure has been incorporated into many breeding programs in Asia (31).

### Contaminations by Synthetic Compounds

**Figure 7:**
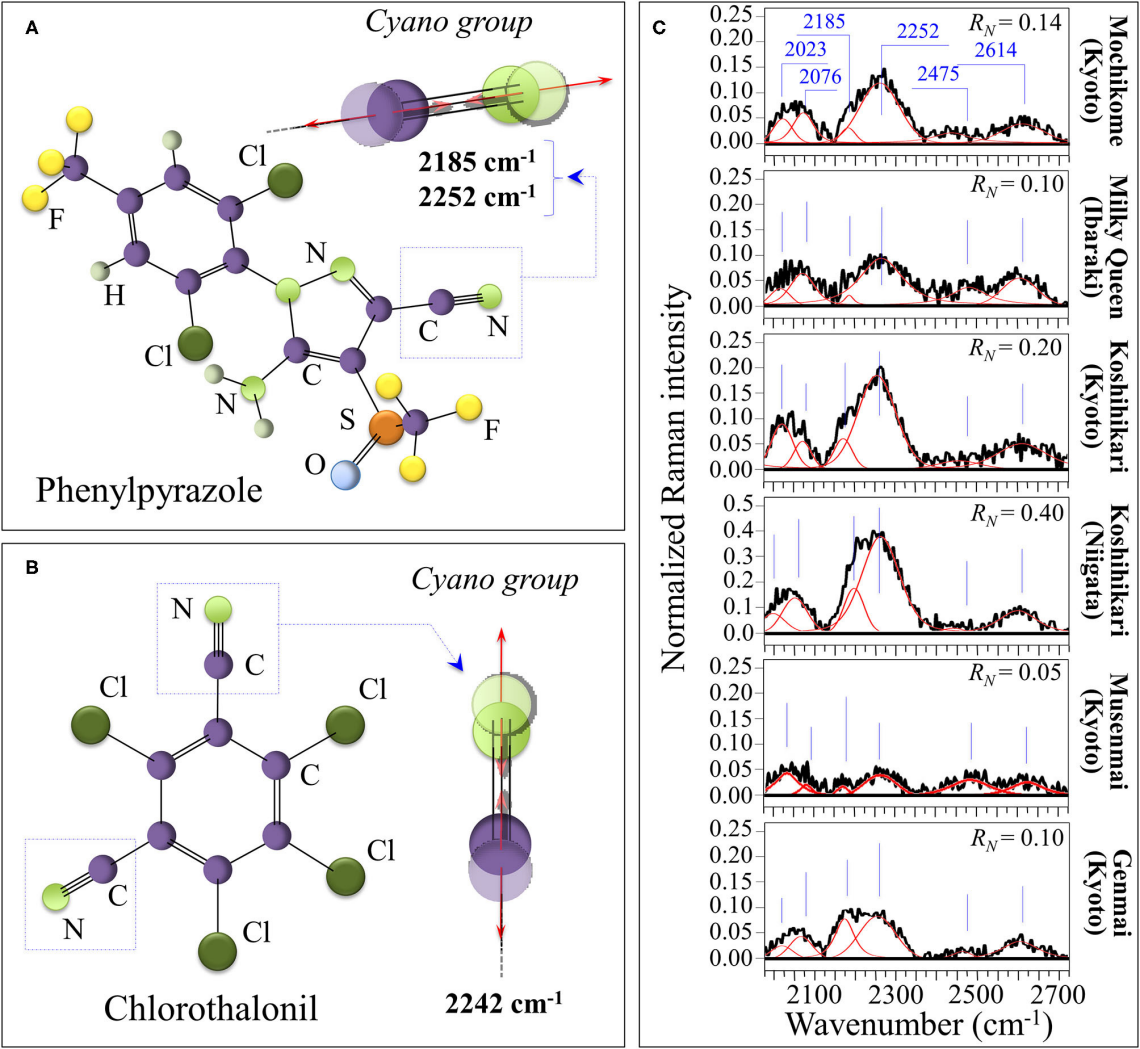
Schematic drafts and C=N stretching vibrations in phenylpyrazole **(A)** and chlorothalonil **(B)** residue from crop pesticides; in **(C)**, Raman spectra of the six investigated cultivars in the spectral region 2,000–2,700 cm^−1^. The clear detection of the doublet at 2,185 and 2,252 cm^−1^ proves the presence of nitrile units, which was quantified according to the contaminant ratio, *R*_*N*_ = *I*_2252_/*I*_478_.

The nitrile (or cyano) group, –C=N, is a functional group with a linear configuration, which reflects the *sp* hybridization of its carbon triple bond. Nitriles, which contain the cyano group, are widely used in the agro industry as fungicides, insecticides, herbicides, and biocides, although they have recently created concerns about their contamination and accumulation in the environment (55). The presence of traces of –C=N, and thus of nitriles can efficiently be detected by Raman spectroscopy (56–58). The symmetric stretching of the C=N triple bond is very sensitive to electron density, with aromatic nitriles that include electron-donating substituent on the ring. For this reason, the C=N related stretching vibrations at 2,200 and 2,242 cm^−1^ appears quite efficiently in the Raman spectrum (59). Moreover, the structural details of their bond in a specific compound slightly alter the spectral location of the high-frequency cyano-group stretching vibrations, thus allowing to guess the type of compound used. More specifically, stretching vibrations for C=N in phenylpyrazole in Figure 7A are seen at 2,185 and 2,252 cm^−1^, while in chlorothalonil in Figure 7B the prominent signal is seen at 2,242 cm^−1^ (56–59). Moreover, the band at 2,252 cm^−1^ could also be contributed by stretching in cyanates, –[O=C=N]^−^. Nitrile and isocyanate groups are both widely used in the manufacture of crop pesticides. Figure 7C shows Raman spectra collected on the six investigated cultivars in the spectral region 2,000–2,700 cm^−1^. The clear detection of the doublet at 2,185 and 2,252 cm^−1^ proves the presence of nitrile units. Accordingly, an estimate of the level of contaminant could be made based on the Raman intensity ratio, *R*_*N*_ = *I*_2252_/*I*_478_, which is proportional to the amount of C=N residues in rice kernels. This spectroscopic parameter is henceforth referred to as contaminant ratio. The lowest *R*_*N*_ value was recorded in the “*Musenmai Kyoto*” cultivar (*R*_*N*_ < 0.05), as expected from the pre-washing procedure, while the highest ones were found in the Koshihikari (Niigata) (*R*_*N*_ ~ 0.4) followed by the Koshihikari (Kyoto) (*R*_*N*_ ~ 0.2). Additional bands were detected at 2,023 cm^−1^, which could be related to diazo compounds (e.g., >C=N^+^=N^−^ group in clofentezine), and at 2,076 cm^−1^, likely related to ketenimines (e.g., >C=C=N– groups in triaryl ketenimines). On the other hand, it is difficult to single out a unique vibrational origin for the two additional bands observed at 2,475 and 2,614 cm^−1^ (cf. Figure 7C). These additional high-frequency bands, which were common to all investigated cultivars, might arise from different types of bond; for example, N–H stretching in quaternary amines or S–H stretching in hydrogen bonded dithioacids –CS–SH. Accordingly, we could not univocally assign them to any specific molecule.

## Discussion

### Glycemic Impact of Different Rice Cultivars

The amount of consumption of dietary carbohydrates is directly related to blood sugar level, which is in turn measured by the glycemic index (GI). Accordingly, rice genotypes are classified according to their rapidly or slowly available glucose levels, their impact on GI widely varying between ~54 and 121 (60). Rice genotypes with low/medium glycemic impact (classified as <69) could allow an improved glycemic control in diabetics and thus greatly reduce the probability of the related cardiovascular diseases (61, 62). A prompt and accurate identification of the GI of rice could play a major role in managing those diseases in an inexpensive way.

**Figure 8:**
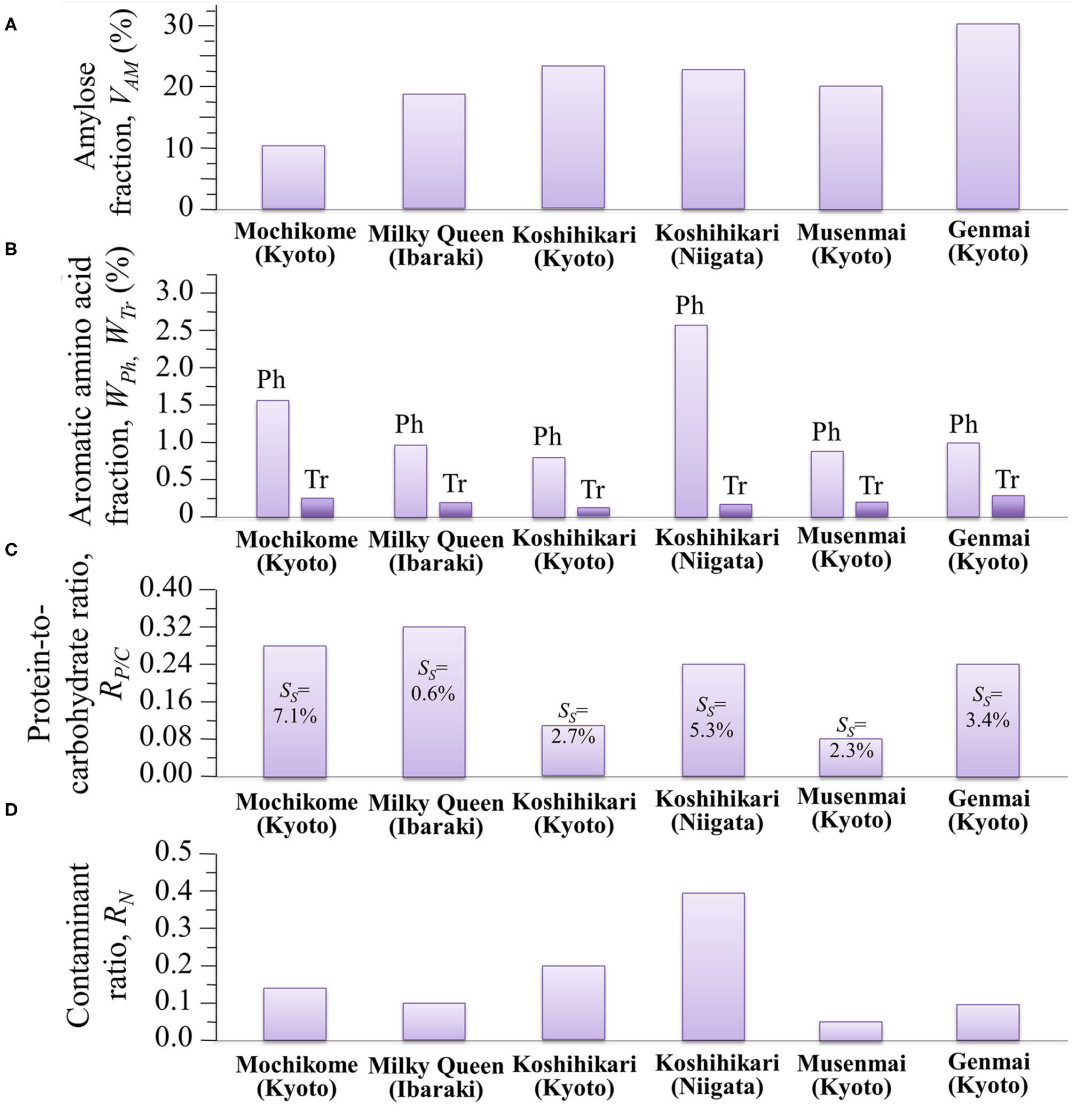
Summary of different nutritional and contamination characteristics as measured by Raman spectroscopy in different Japanese cultivars: **(A)** amylose volume fraction, *V*_*AM*_ (%); **(B)** aromatic amino acid volume fractions, *W*_*Ph*_ (%) and *W*_*Tr*_ (%) (the abbreviations Ph and Tr stem for phenylalanine and tryptophan, respectively); **(C)** protein-to-carbohydrate ratio, *R*_*P*/*C*_, with succinylation fractions, *S*_*S*_, in inset; and, **(D)** contaminant ratio, *R*_*N*_.

The glycemic impact of a rice cultivar is inversely proportional to its amylose content, the higher the amylose content the lower the GI, and vice versa. This relationship arises from variations in the degree of gelatinization, which represents the propensity of the carbohydrate structures to break down into elementary glucose rings that are easily transported into the blood stream. Since the C1-O-C6 branching bonds of amylopectin are more promptly cleaved as compared to the C1-O-C4 bonds of amylose chains, amylopectin-rich rice cultivars promote gelatinization and maximize GI. Inversely, rice cultivars rich in amylose are less susceptible to gelatinization and minimize GI. Assessments of amylose content by the present Raman experiments revealed volumetric fluctuations in amylose content up to 300% among different cultivars. The amylose volume fractions, *V*_*AM*_ (%), measured in different cultivars are summarized in Figure 8A. According to Jeevetha et al. (63), GI is a linear function of *V*_*AM*_ (%) and a decrease of ~5 % in *V*_*AM*_ leads to a jump of ~20 points in GI. Using such an estimate, it is clear that the glycemic impact of Milky Queen rice brand, which is lower than those of both Koshihikari cultivars by ~5 vol.%, induces a significantly higher glycemic impact (~20 points in GI).

In addition to genomic impact (primarily controlled by the so-called *Waxy* gene coding for granule bound starch synthase) (64), the amylose content of samples of the same cultivar might also fluctuate up to six points percent; the major environmental factor being the ambient temperature (local climate) during grain development (65, 66). A low ambient temperature increases amylose content and lowers gelatinization propensity, while a high ambient temperature has the opposite effect. Such fluctuations have a definitely not negligible impact when it comes to eating habits or to selecting a diet.

The present study provides clear evidence of the wide variety of carbohydrate structures contained in commercially available Japanese cultivars. The Raman evaluation is comprehensive of gene coding, environmental factors, growing, and aging conditions.

### Aromatic Amino Acids, Protein Contents and Other Nutritional Facts

Aromatic amino acids represent the building blocks of proteins and are important precursors of secondary metabolism. Phenylalanine and tryptophan, although representing essential molecules for the human metabolism, cannot be synthesized in the human body and their supply depends on nutritional provision. Modified biosynthetic pathways have thus been searched for in order to accumulate selected amino acids in rice at high concentrations (5, 31, 50). Figure 8B summarizes our spectroscopic quantifications of weight fraction of phenylalanine and tryptophan in the six types of studied cultivars. Also in the case of aromatic amino acids, similar to the case of amylose contents, we found significant differences among different rice cultivars, which might be associated with the production conditions (including climate, mineral, nutrition, etc.); with the highest amount of phenylalanine and tryptophan being recorded in Koshihikari (Niigata) and Genmai (Kyoto), respectively. Despite its moderate amount of aromatic amino acids, the low amylose cultivar Milky Queen scored the highest protein-to-carbohydrate ratio (*R*_*P*/*C*_ = 0.32) (cf. Figure 8C), which was ~14% higher than that of glutinous rice. While the intensity of phenylalanine ring breathing band at 1,004 cm^−1^ is generally accepted as indicative of protein contents in food analysis (67), the present data suggest that this criterion cannot be applied to the analysis of protein content in rice cultivars. A significant difference was detected between the two Koshihikari brands, the one from Niigata Prefecture being more than twice richer in proteins than the one from Kyoto. The protein fraction in the Koshihikari (Niigata) was comparable with that of the Genmai brand, while, as expected the Musenmai type was the lowest in protein content as a consequence of the polishing procedure. The four constituents of rice protein are albumin, globulin, glutelin and prolamin (68). Among these proteins, the richest in phenylalanine is glutelin (~12.6 g/100 g protein), followed by prolamin (~10.7 g/100 g protein). Conversely, albumin is containing the lowest amount (~3.8 g/100 g protein), followed by globulin (~5.3 g/100 g protein) (69). Based on these considerations, the Milky Queen brand, which is the richest one in proteins but only contains a moderate fraction of phenylalanine, is likely mainly containing albumin and globulin. Conversely, the Koshihikari (Niigata) brand, which is the richest in phenylalanine but contains a lower amount of proteins, might be richer in glutelin and prolamin. Note also that the Koshihikari (Niigata) was also the cultivar showing the highest degree of succinylation (cf. *S*_*S*_ values given in inset to Figure 8C). Substantial succinylation in this type of rice is likely applied in order to obtain improved kernel clarity, low temperature stability, and reduced retrogradation tendency (70).

Although not directly related to the nutritional value of the rice cultivar, the evaluation of the fraction of synthetic contaminants represents an important parameter in judging the quality of a selected rice brand. Figure 8D summarizes the spectroscopic findings in terms of contaminant ratio, *R*_*N*_, whose values have been reported in section Contaminations by Synthetic Compounds. We have located the nitrile band in the Raman spectra of all the investigated Japanese cultivars (cf. Figure 7C). Two popular herbicides/fungicides that contain nitrile groups were drawn in Figures 7A,B. The former draft represents the phenylpyrazole (4-chloro-2-cyano-N,N-dimethyl-5-p-tolylimidazole-1-sulfonamide) molecule. This molecule has been used in Japan since 1987, but it is now subjected to a strict regulation allowing its usage only in the initial seedling stage of in-box plant growth, in order to avoid its dispersion in the environment. On the other hand, the latter draft represents the chlorothalonil (2,4,5,6-tetrachloroisophthalonitrile) molecule; the environmental effect of this molecule was reviewed in 2019 and found to involve high risks to amphibians and fishes since chlorothalonil breakdown products may cause DNA damage. The European Commission decided not to renew this fungicide. However, in Japan, the use of products containing chlorothalonil is allowed although only in the initial seedling stage of in-box plant growth. The present Raman experiments newly revealed that nitrile-group containing molecules, although only used in the initial stage of plant growth, yet stem in the final rice kernel products.

### The Raman Barcode Algorithm

Facilitating real-time data access and exchange is an essential step in tracking food quality and safety. However, the present practices of traceability, tracking, and record keeping of product flow from production to supply usually rely on a limited set of variables and do not provide technical information about quality and safety of food products. For this reason, the development of more effective tools of record keeping and information sharing could represent a suitable step forward. We propose here to interface a barcode with Raman spectroscopic assessments that rapidly and factually measure rice nutritional quality and monitor possible contaminations from external agents (e.g., herbicides/fungicides). The main advantages in using a Raman spectroscope reside in the possibility of quickly and non-destructively measuring at once a number of different characteristics, as demonstrated in this paper. Barcodes, which enable electronic recordkeeping and quick accessibility/readability to users through apps and user-friendly software, could be matched to the Raman spectrum in a number of different ways. Moreover, the Raman spectroscopic method appears to have both the flexibility and the swiftness necessary to follow variations related to interannual, environmental, and storage conditions.

**Figure 9:**
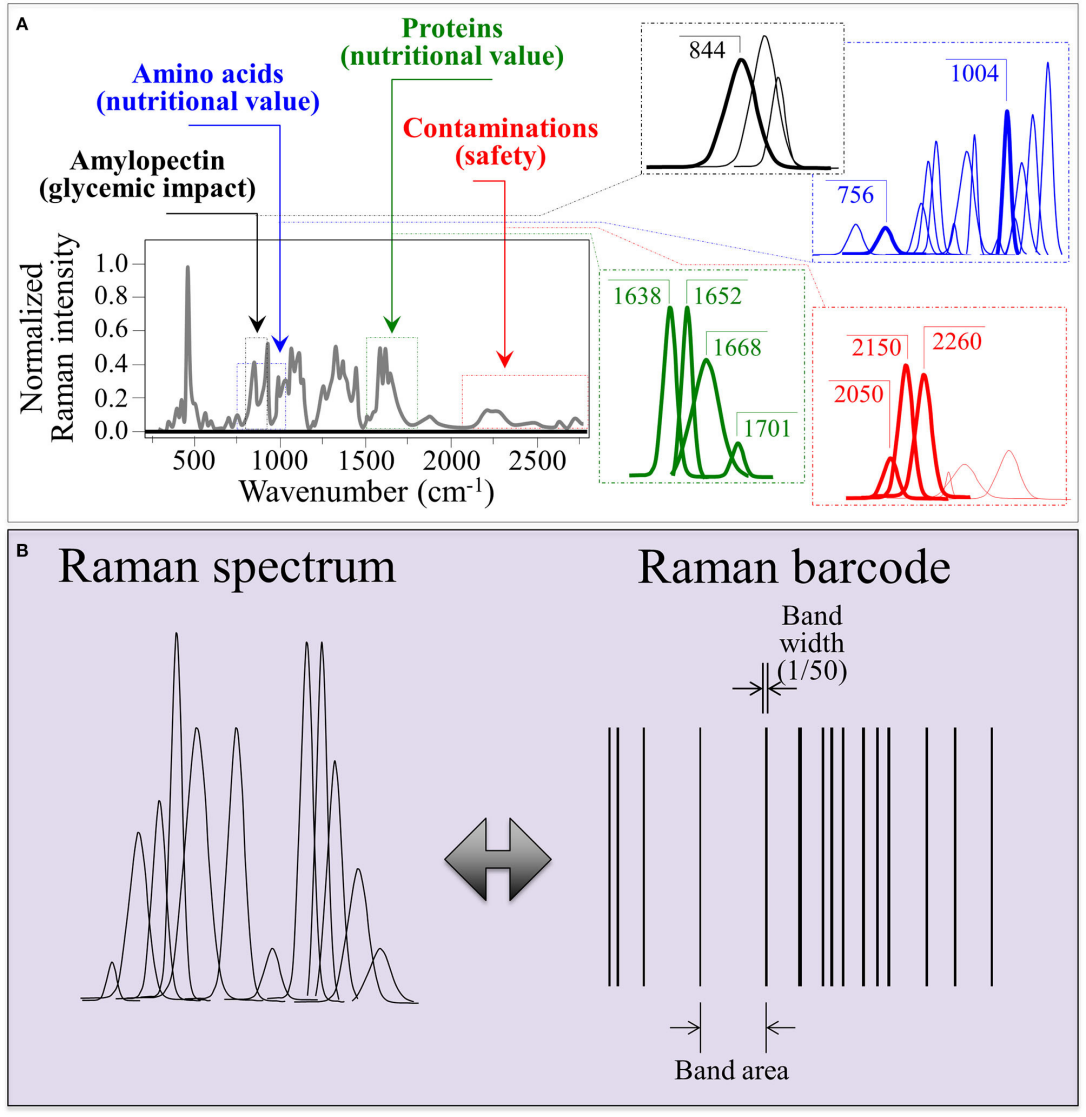
**(A)** Schematic drafts locating different zones of the Raman spectrum representing different nutritional and quality characteristics and their deconvoluted sub-band components; and **(B)**, a proposed algorithm to convert the sub-band sequence into a linear barcode.

**Figure 10:**
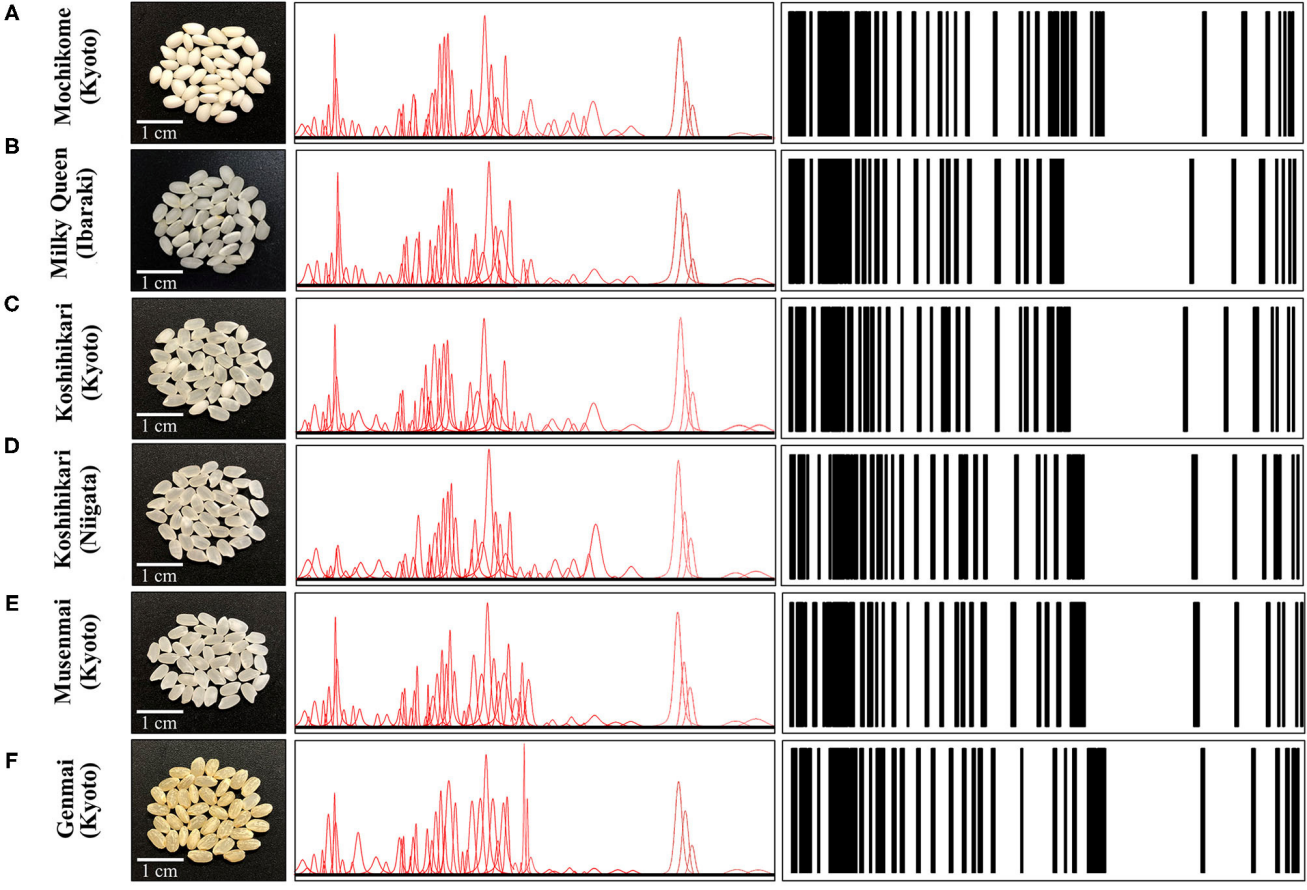
Laser microscopy images of kernel cross sections from six different Japanese rice cultivars **(A)** Mochikome (Kyoto), **(B)** Milky Queen (Ibaraki), **(C)** Koshihikari (Kyoto), **(D)** Koshihikari (Niigata), **(E)** Musenmai (Kyoto), and **(F)** Genmai (Kyoto). Photographs of the kernels of six popular Japanese rice cultivars (cf. insets), the Voigtian sub-band sequences derived from their related Raman spectra, and the computed barcodes according to the algorithm shown in Figure 9B.

As an example of Raman spectrum/barcode matching in rice products, Figure 9 drafts one of those possible ways to generate a barcode from the Raman spectrum of rice. In Figure 9A, different zones of the Raman spectrum, which represent glycemic impact, nutritional value in terms of aromatic amino acids and protein contents, and the amount of herbicide/fungicide contamination, are located and deconvoluted into individual sub-band components. In Figure 9B, an algorithm converts the sub-band sequence in the full Raman spectrum into a barcode upon assigning to each band a line with thickness = 1/50 of the sub-band width and a distance from the successive line proportional to the band area. Each line in the barcodes corresponds to a specific peak in the Raman spectrum. Although the barcode built with the above criterion univocally locate the nutritional quality of the rice cultivar, the critical step in this analysis relates to sampling, namely, the number of spectra collected to build up the average spectrum assumed as representative of the entire rice cultivar; the higher the number of spectral measurements, the more accurate the result. In Figures 10A–F, deconvolutions into sub-bands and related barcodes are shown for average Raman spectra recorded on different rice cultivars. The “Raman barcodes” should then be decrypted into easily readable nutritional and quality information through appropriate apps. The authors believe that this approach, once developed into capillary networks, could bring in an important contribution in managing information about food quality and safety in our modern society.

Ziegler and Patel have previously proposed Raman barcodes (71). Unlike the model of barcode put forward in this study, their algorithms are based on the second derivative of the Raman spectra. The advantage of Ziegler's barcode is flexibility, as it can be used to represent any data array, up to a technically unlimited number of bands. Our approach, on the other hands, uses a fixed number of known Raman bands, increasing repeatability and consistency for specific products.

## Conclusion

Raman spectroscopy is emerging as a powerful analytical technique in food science because of its flexibility, promptness, non-contact, and non-destructiveness in evaluating food quality and nutritional facts. This offers exciting opportunities for enhancing food safety and quality, as well as in systematically and thoroughly controlling health-related issues. We have shown here how the Raman technology could unfold in single measurement key nutritional issues, such as the fractions of dietary carbohydrates, the concentration of antioxidant aromatic amino acids, and the structure/fraction of proteins including important details such the fraction of succinylation and the presence of carotenoids. Advancements in Raman technology stimulate researchers to think outside the box and to meet new challenges. It is hoped that the conceptual framework described in this paper could stimulate multidisciplinary research efforts bringing together researchers in food science, biotechnology, chemistry, and information technology, thus unfolding fundamental issues related to consumers' health and satisfaction.

## Data Availability Statement

The raw data supporting the conclusions of this article will be made available by the authors, without undue reservation.

## Author Contributions

Y-IS, TN, and TM contributed to conception and design of the study. WZ organized the database. WZ and GP performed the statistical analysis and molecular modeling. HC, EM, and FB carried out the experiments. GP wrote the first draft of the manuscript. All authors contributed to manuscript revision, read, and approved the submitted version.

## Conflict of Interest

The authors declare that the research was conducted in the absence of any commercial or financial relationships that could be construed as a potential conflict of interest.
