# Supplementary material for: Raman Molecular Fingerprints of Rice Nutritional Quality and the Concept of Raman Barcode
Source: Front Nutr. 2021 Jun 23;8:663569. doi: 10.3389/fnut.2021.663569 (PMC8260989; doi:10.3389/fnut.2021.663569)
Supplement: Supplementary file 1 [file Data_Sheet_1.pdf]

Supplementary Material

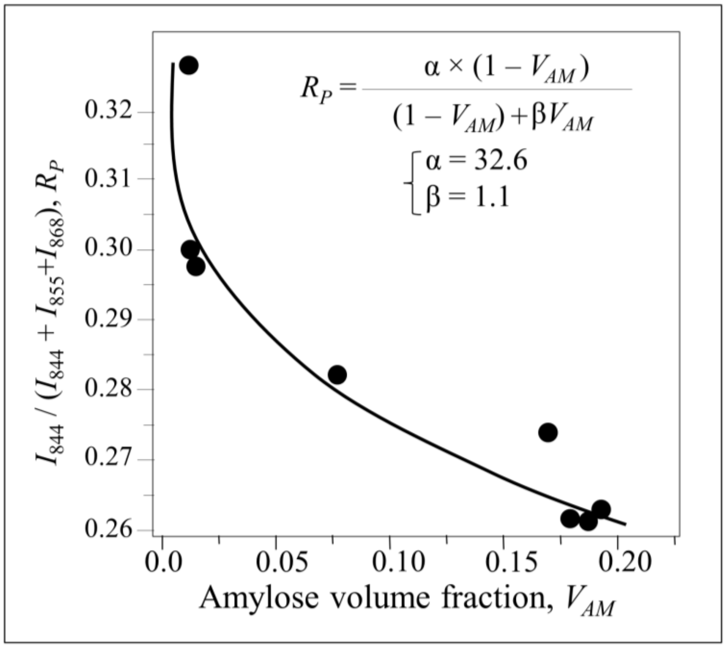

Supplementary Figure 1. Calibration plot and best fitting curve to retrieve the numerical calibration constants α and β in Eq.(1), needed for computing the amylose volume fraction, V_{AM}, from Raman relative intensity ratios. Data were fitted according to a least-square procedure.

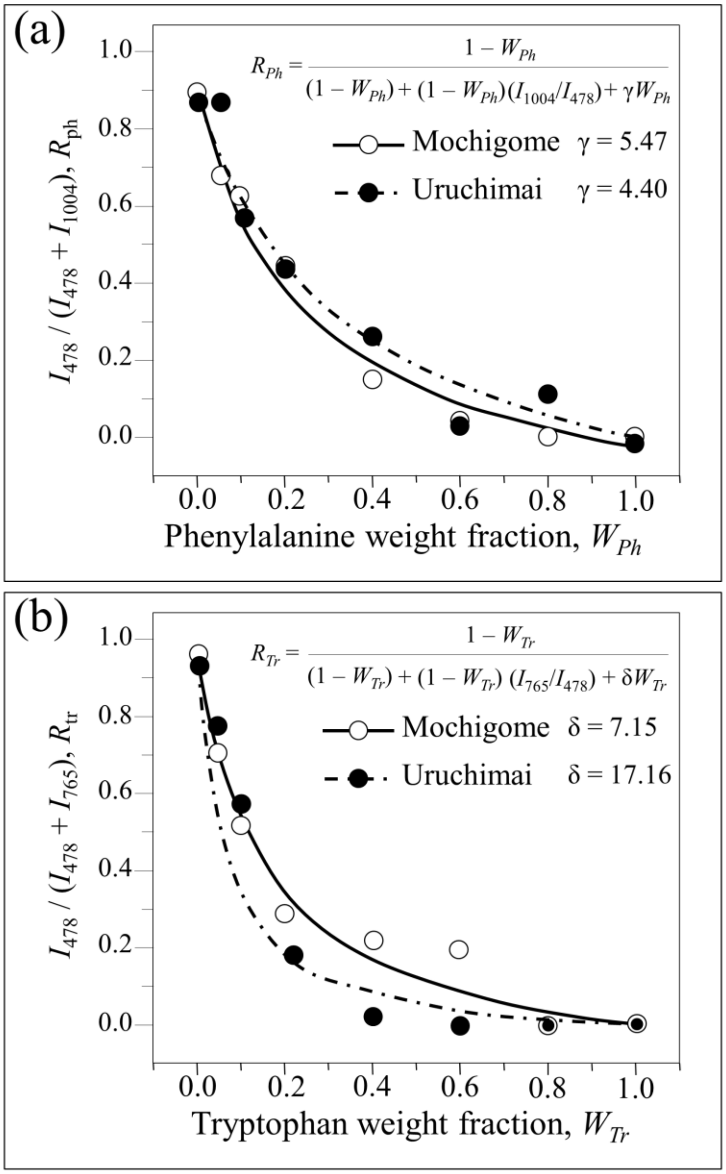

Supplementary Figure 2. Calibration plots and best fitting curves to retrieve the numerical calibration constants γ and δ in Eqs.(4) and (6), needed for computing (a) the phenylalanine and (b) the tryptophan weight fractions, W_{Ph} and W_{Tr}, respectively, from Raman relative intensity ratios. Data were fitted according to a least-square fitting procedure and the retrieved constant differed for glutinous (Mochigome) and non-glutinous (Uruchimai) types of rice kernels (cf. values in inset).

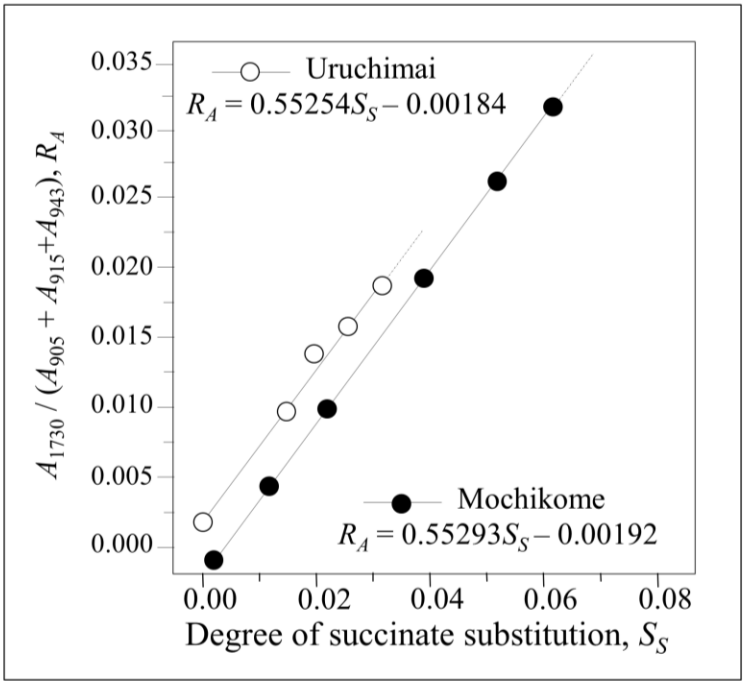

Supplementary Figure 3. Calibration plot and best fitting lines to retrieve the dependences needed for computing the degree of succinate substitution, S_s, from Raman relative intensity ratios for glutinous (Mochigome) and non-glutinous (Uruchimai) rice cultivars (in Eqs.(8) and (9), respectively). Data were fitted according to a least-square fitting procedure.
